# Analysis of the association between cholinesterase and in-hospital mortality in children with bloodstream infections in the pediatric intensive care unit

**DOI:** 10.3389/fped.2025.1588634

**Published:** 2025-08-08

**Authors:** Zuoxu Ding, Zijiu Sun, Qi Zhong

**Affiliations:** ^1^ Department of Clinical Laboratory, Shanghai Children's Medical Center, Shanghai Jiao Tong University School of Medicine, Shanghai, China; ^2^ Department of Laboratory Medicine, Shanghai Tenth People’s Hospital, School of Medicine, Tongji University, Shanghai, China

**Keywords:** butyrylcholinesterase enzyme (BChE), pediatric intensive care unit (PICU), bloodstream infection (BSI), children, database

## Abstract

**Objective:**

Our aim was to assess the relationship between BChE levels on admission to the intensive care unit and death from bloodstream infections in the pediatric intensive care unit (PICU).

**Methods:**

We conducted a retrospective analysis using the Pediatric Intensive Care Unit Database (a large Chinese paediatric intensive care database from 2010 to 2018) to assess BChE levels at the time of intensive care unit admission in 329 critically ill children with bloodstream infection admitted to the intensive care unit. We analyzed the relationship between BChE and death from bloodstream infections. We used multifactor logistic analysis regression and adjusted smooth spline plots to estimate the relationship between BChE and death from bloodstream infections.

**Results:**

Of 329 children, 53 (16%) died in hospital. After correction for confounders, BChE was negatively associated with the risk of death in the PICU. For every 1,000 U/L increase in BChE, the risk of death was reduced by 16% (corrected OR = 0.84, 95% CI: 0.79, 0.89). After adjusting for confounders, the risk of death decreased by 23% for every 1,000 U/L increase in BChE (OR = 0.77, 95% CI: 0.63, 0.96). Patients with BChE levels between 5,000 and 8,000 U/L had a 51% lower risk of death, while those with BChE levels >8,000 U/L had a 77% lower risk of death, compared with those with BChE levels <5,000 U/L.

**Conclusion:**

According to multiple regression analysis, decreased BChE is an independent risk factor for all-cause mortality in children with bloodstream infections in pediatric intensive care units.

## Introduction

Bloodstream infection and their complications have a high incidence and mortality rate and can cause a heavy economic burden (1, 2). Primary bloodstream infection (BSI), a major and avoidable infectious complication, significantly affects the prognosis of critically ill patients. Gray et al. (3) published a 3-year survey of bacteremia in a pediatric intensive care unit (PICU). The study found that for every 1,000 admissions, there were 39.0 instances of infection. The majority of these infections (64.1%) originated in the intensive care unit, while a smaller portion (20.6%) was contracted outside the hospital. The remaining 15.3% of infections occurred in various other hospital areas. The crude mortality rate among children with BSIs was significantly higher at 26.5%, compared with 8.1% among those without BSI. Approximately 1.2 million children worldwide contract sepsis every year, with a 1%–5% mortality rate overall and 9%–20% in severe cases (4).

Serum cholinesterase, also known as pseudocholinesterase or butyrylcholinesterase (BChE), is present in serum and synthesized (5, 6). It has been established in previous research that a reduced activity of BChE is correlated with an unfavorable prognosis in cases of bloodstream infection (7–10). In addition, BChE activity reduction is related to the mortality of critically ill patients (11–18). According to the Third International Consensus Definitions for Sepsis and Septic Shock (Sepsis-3), sepsis is characterized as a complex condition that encompasses a range of physiological, pathological, and biochemical abnormalities triggered by an infection (19). Sepsis is a critical and potentially lethal illness that significantly impacts global public health (20, 21). Early diagnosis and active intervention for patients with bloodstream infection can decrease mortality rates and are of significant clinical importance (22).

Several studies report that BChE activity is markedly reduced when adults or children are admitted with sepsis or septic shock, a decline attributed to both impaired hepatic synthesis and enzyme consumption during systemic inflammation (23, 24). BChE levels are easily measurable in a routine blood test. However, the significance of these levels in diagnosing and forecasting the progression of sepsis is still uncertain (25). Although the topic has received some attention, studies examining pediatric intensive care unit patients remain scarce. In this study, we will investigate the BChE levels of pediatric intensive care unit patients at admission, evaluate the relationship between BChE and bloodstream infection mortality, and determine whether lower BChE activity is associated with bloodstream infection mortality in such patients.

## Methods

### Subjects

This retrospective study was conducted using the Pediatric Intensive Care (PIC) database, a publicly available clinical dataset designed specifically for pediatric research. The PIC database contains detailed information on critically ill children, including laboratory results, length of hospital stay, and survival outcomes.

The PIC database was accessed in accordance with data use protocols outlined by the PIC website and PhysioNet. The study was approved by the Ethics Committee/Institutional Review Board of the Children's Hospital affiliated with Zhejiang University School of Medicine. As the research was retrospective and did not affect clinical care, the need for individual patient consent was waived. All patient data were anonymized to protect privacy (26). The data were collected from patients admitted to the PICU of the Children's Hospital, which is affiliated with the Zhejiang University School of Medicine in China. We included 329 critically ill children who had complete laboratory test data available from the first examination conducted within 24 h of PICU admission.

Exclusion criteria were as follows: missing or incomplete laboratory tests within the first 24 h of PICU admission; blood cultures that were either negative or yielded only coagulase-negative Staphylococci; blood samples collected outside the predefined time window; extreme outlier values or missing key variables; and admission to the neonatal intensive care unit.

### Statistical analysis

All analyses were performed using the R and SPSS, with a value of *p* < 0.05 considered statistically significant. Quantitative data are presented as mean ± standard error of mean, median and interquartile range, and ranges depending on the distribution of the data. Risk factors were evaluated in univariate analysis and by multivariate analysis by a multiple logistic regression procedure. Multivariable logistic regression models were constructed to assess the independent association between BChE levels and all-cause mortality. Model adjusted for clinical indicator, including age, gender, and ICU category, creatinine, albumin, hemoglobin, alanine aminotransferase, neutrophil, lactate, and total cholesterol, which are potential confounders based on clinical plausibility and univariate analysis. Logistic regression models were used to examine the effect of BChE on mortality from bloodstream infections. A multivariate logistic regression model was used to identify risk factors for mortality caused by BSI and then a smooth curve fitting was built to investigate their internal nonlinear relationships. The risk associated with mortality is reported per 1,000 U/L of continuous BChE. We determined and grouped the best cutoff points for survival analysis.

## Results

There were 329 records included in this study. Of all patients included, 14.6% (*n* = 48) were recruited in the SICU, 33.7% (*n* = 111) in the PICU, 31% (*n* = 102) in the General ICU, and 20.7% (*n* = 68) in the CICU. In the gender distribution of patients, 83.9% were female (*n* = 276). Mortality factors are associated with the following variables: BChE (OR 0.78, 95% CI 0.67–0.91, *p* < 0.001), creatinine (OR 1.02, 95% CI 1.01–1.03, *p* < 0.001), cholesterol (OR 0.70, 95% CI 0.52–0.94, *p* = 0.016), lactate (OR 1.27, 95% CI 1.14–1.40, *p* < 0.001), and neutrophil (OR 1.11, 95% CI 1.04–1.17, *p* < 0.001). Compared with the group that did not survive, it was more prevalent in the survival group.

**Figure 1:**
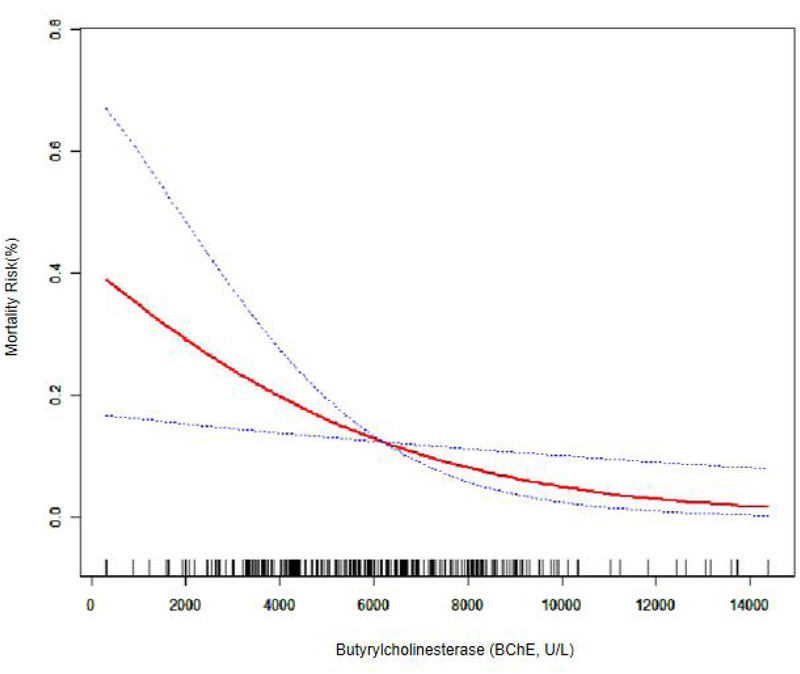
Curve fitting of BChE.

Table 1 describes the baseline characteristics of the subjects, including demographic characteristics and the results of a number of laboratory tests that may be associated with the occurrence of death. Table 2 shows the results of multiple regression of BChE on the risk of death. Table 3 shows the results of multiple regression of BChE on the risk of death. Without adjusting for confounding factors, the risk of death was reduced by 22% for every 1,000 U/L rise in BChE (OR = 0.78, 95% CI: 0.67, 0.91). With full correction for confounders, the risk of death was reduced by 23% for every 1,000 U/L rise in BChE (OR = 0.77, 95% CI: 0.63, 0.96); and BChE was grouped according to thresholds of 5,000 and 8,000, and after fully adjusting for confounders, there was a statistically significant 51% reduction in the risk of death in patients with BChE between 5,000 and 8,000 U/L (OR = 0.49, 95% CI: 0.26, 0.94) and a 77% reduction in the risk of death in those with BChE >8,000 U/L, (OR = 0.23, 95% CI: 0.07, 0.71) compared with those with BChE <5,000 U/L. According to curve fitting (Figure 1), mortality decreased as BChE rose.

**Table 1:** Baseline characteristics of neonates with bloodstream infection–related mortality and those who survived their bloodstream infection episode.

Variables	Survived (*N* = 276)	Mortality (*N* = 53)	*P*-value
Age (months)	10.62 (3.38–47.45)	6.44 (2.27–36.92)	0.307
ALT (U/L)	60.13 (111.74)	85.46 (137.47)	0.452
Cr (µmol/L)	48.15 (21.94)	66.60 (48.57)	0.057
Tcho (mmol/L)	3.16 (1.07)	2.74 (1.29)	0.011
Hb (g/L)	109.60 (23.15)	112.08 (30.73)	0.934
SaO_2_ (mmHg)	89.16 (14.84)	86.91 (16.07)	0.253
LAC (mmol/L)	2.57 (2.14)	4.54 (3.60)	<0.001
Neutrophil (×10^9^/L)	5.73 (4.43)	8.33 (5.69)	0.005
Cholinesterase (U/L)	6,160.60 (2,414.87)	5,000.79 (2,120.26)	<0.001
Gender, *N* (%)			
Female	110 (39.86%)	17 (32.08%)	0.287
Male	166 (60.14%)	36 (67.92%)	
FIRST_CAREUNIT			
SICU	43 (15.58%)	5 (9.43%)	0.013
CICU	62 (22.46%)	6 (11.32%)	
PICU	95 (34.42%)	16 (30.19%)	
General ICU	76 (27.54%)	26 (49.06%)	

ALT, alanine aminotransferase; Cr, creatinine; Tcho, total cholesterol; LAC, lactate.

**Table 2:** Risk factors of bloodstream infection.

Risk factor	95% CI	*P*-value
Cholinesterase (U/L)	0.78 (0.67, 0.91)	<0.001
Age (months)	1.00 (0.99, 1.00)	0.620
ALT (U/L)	1.00 (1.00, 1.00)	0.167
Cr (µmol/L)	1.02 (1.01, 1.03)	<0.001
Tcho (mmol/L)	0.70 (0.52, 0.94)	0.016
Hb (g/L)	1.00 (0.99, 1.02)	0.506
SaO_2_ (mmHg)	0.99 (0.97, 1.01)	0.331
LAC (mmol/L)	1.27 (1.14, 1.40)	<0.001
Neutrophil (×10^9^/L)	1.11 (1.04, 1.17)	<0.001
Gender, *N* (%)		
Female	127 (38.60%)	1.000
Male	202 (61.40%)	0.288
FIRST_CAREUNIT		
SICU	48 (14.59%)	1.000
CICU	68 (20.67%)	0.773
PICU	111 (33.74%)	0.496
General ICU	102 (31.00%)	0.039

**Table 3:** Individual effect of BChE on all-cause mortality.

Exposure	Non-adjusted		Adjust I		Adjust II	
	OR (95% CI)	*p*-value	OR (95% CI)	*p*-value	OR (95% CI)	*p*-value
Continuous cholinesterase per 1,000 U/L	0.78 (0.67, 0.91)	<0.001	0.78 (0.66, 0.91)	0.002	0.77 (0.63, 0.96)	0.017
Clinical cutoffs						
<5,000	Reference		Reference		Reference	
≥5,000, <8,000	0.52 (0.28, 0.96)	0.038	0.49 (0.26, 0.94)	0.031	0.54 (0.22, 1.32)	0.176
≥8,000	0.24 (0.08, 0.72)	0.011	0.23 (0.07, 0.71)	0.011	0.16 (0.03, 0.74)	0.018

Adjust model I: Adjusted for age, intensive care unit category, gender. Adjust model II: Adjusted for gender, age, intensive care unit category, Cr, Alb, hemoglobin, ALT, absolute neutrophil, LAC, TG, Tcho.

## Discussion

This research, utilizing the PIC database, conducted a challenging and innovative analysis of the correlation between BChE and in-hospital mortality in children with bloodstream infections in the PICU. Our study clarified the relationship between BChE levels and mortality in children with bloodstream infections in the PICU. Also, we demonstrated that reduced BChE was an independent risk factor for the risk of death from bloodstream infections in the PICU, after adjusting for age, type of intensive care unit, neutrophils, albumin, ALT, and Cr. Bloodstream infection is an important cause of death in critically ill patients. Lower BChE activity in pediatric patients with sepsis is associated with increased mortality in the PICU setting.

This retrospective analysis leveraged the Pediatric Intensive Care (PIC) database to explore the association between BChE and mortality in children with BSI admitted to the PICU. Our study clarified the relationship between BChE levels and mortality in children with bloodstream infections in the PICU. We demonstrated a clear inverse association between BChE levels measured within 24 h of the first positive blood culture and in-hospital mortality, independent of established confounding factors including age, gender, ICU type, and standard clinical parameters (e.g., neutrophils, albumin, alanine, and creatinine). Bloodstream infection is an important cause of death in critically ill patients. However, due to the non-specific symptoms of bloodstream infection, the ability to accurately identify postintervention is critical to improving bloodstream infection outcomes. In previous studies, lower BChE activity has been associated with adverse clinical outcomes across various critical conditions (27, 28). There are not many studies on acetylcholinesterase activity to predict sepsis outcome and the mechanism is not clear. Also, it is notable that studies of cholinesterase have largely focused on adults rather than critically ill children. This study is an important proof and addition to the field; moreover, it fills the gap of this study in the population of children.

Bahloul et al. (29) conducted a one-year prospective, single-blinded study in which nearly all patients admitted to the ICU presented with elevated C-reactive protein (CRP) and procalcitonin (PCT) levels alongside reduced BChE activity. Their findings demonstrated that BChE serves as a reliable predictor for septic shock caused by bacterial infections, outperforming traditional inflammatory markers such as CRP and PCT. Similarly, Tobias et al. (24) provided supportive evidence through a prospective study of 239 adult patients with sepsis or septic shock, where multivariable regression analysis showed that patients with cholinesterase activity below the cohort median had a significantly higher risk of 30-day all-cause mortality. Yue et al. (30) reported an inverse link between admission BChE and mortality in a broad PICU population, whereas our study narrows the focus to children with culture-confirmed bloodstream infection, samples cholinesterase within a strictly defined early window, and demonstrates a clear dose response with outcome.

Collectively, these studies highlight that patients with sepsis typically exhibit persistently low BChE activity, underscoring its prognostic relevance in critically ill populations. Furthermore, emerging research has demonstrated the prognostic significance of BChE activity in other severe clinical conditions. For instance, Harrington et al. (31) identified decreased BChE activity as a potential biomarker for sudden infant death syndrome, linking it to autonomic dysfunction. Markuskova et al. (32) found that reduced BChE activity in COVID-19 patients was not only associated with poorer outcomes but also inversely correlated with inflammatory markers such as IL-6 and CRP. Recently, the same group further confirmed these findings that highlighting BChE activity consistently reflects the inflammatory status and may serve as a prognostic biomarker for critical illness beyond COVID-19 (33). This new evidence reinforces the broader relevance of BChE as an indicator of systemic inflammation and adverse outcomes.

These findings reinforce the value of BChE as a novel prognostic biomarker reflecting both inflammatory status and clinical outcomes and further suggest its potential utility in high-risk pediatric populations. Researchers have also discussed the possible mechanisms behind the decrease in cholinesterase activity in patients with blood infection. Loss caused by increased capillary permeability is one of the main mechanisms for BChE reduction in patients with blood infection (34). Recent studies have implicated the cholinergic anti-inflammatory pathway (CAP)—which operates through vagal acetylcholine release and its interaction with α7 nicotinic acetylcholine receptors—in the modulation of systemic inflammation during sepsis. Although the precise role of BChE in this pathway remains unclear, its systemic presence and acetylcholine-hydrolyzing activity suggest a potential regulatory role in peripheral cholinergic signaling. Moreover, cholinesterase inhibitors have been observed to exert anti-inflammatory properties through their interaction with the cholinergic anti-inflammatory pathway, as evidenced by studies conducted on sepsis in animal models (35–38).

To our knowledge, this is one of the few clinical studies evaluating the prognostic role of BChE in a pediatric cohort of bloodstream infections, highlighting the potential role of BChE as a biomarker for the identification of early bloodstream infections in critically ill children. However, this study has several limitations. First, the sample size of this study was small, and the current sample size does not allow for further subgroup analyses, which need to be further increased for validation. Second, the single-center, observational nature of our study may introduce unmeasured confounders, despite our efforts to adjust for them with multiple regression analysis. In addition, this study has yet to explain the mechanism of low BChE activity in children with septicemia, which requires further experimental research. Another point to discuss is the challenge of precisely identifying sepsis incidents in non-experimental studies, a task that becomes even more difficult when dealing with children. Furthermore, due to the lack of detailed medication data in the PIC database, we could not adjust for the potential effects of drugs known to inhibit BChE activity, which may represent an additional source of residual confounding.

## Conclusion

According to multiple regression analysis, decreased BChE is an independent risk factor for all-cause mortality in children with bloodstream infections in pediatric intensive care units. For children admitted with a suspected diagnosis of bloodstream infection, we can intervene in a timely manner based on their cholinesterase status.

## Data Availability

Publicly available datasets were analyzed in this study. This data can be found here: https://doi.org/10.13026/32x9-wv38.
